# FGFR3, as a receptor tyrosine kinase, is associated with differentiated biological functions and improved survival of glioma patients

**DOI:** 10.18632/oncotarget.13139

**Published:** 2016-11-05

**Authors:** Zheng Wang, Chuanbao Zhang, Lihua Sun, Jingshan Liang, Xing Liu, Guanzhang Li, Kun Yao, Wei Zhang, Tao Jiang

**Affiliations:** ^1^ Beijing Neurosurgical Institute, Capital Medical University, Beijing, China; ^2^ Beijing Tiantan Hospital, Capital Medical University, Beijing, China; ^3^ Sanbo Brain Hospital, Capital Medical University, Beijing, China; ^4^ Center of Brain Tumor, Beijing Institute for Brain Disorders, Beijing, China; ^5^ China National Clinical Research Center for Neurological Diseases, Beijing, China; ^6^ Chinese Glioma Genome Atlas network (CGGA), Beijing, China

**Keywords:** FGFR3, receptor tyrosine kinase, FGFR-TACC fusion genes, glioma

## Abstract

**Background:**

Activation of receptor tyrosine kinases is common in Malignancies. FGFR3 fusion with TACC3 has been reported to have transforming effects in primary glioblastoma and display oncogenic activity *in vitro* and *in vivo*. We set out to investigate the role of FGFR3 in glioma through transcriptomic analysis.

**Results:**

FGFR3 increased in Classical subtype and Neural subtype consistently in CGGA and TCGA cohort. Similar patterns of FGFR3 distribution through subtypes were observed in CGGA and TCGA samples. Gene ontology analysis was performed with genes that were significantly correlated with FGFR3 expression. We found that positively associated biological processes of FGFR3 were focused on differentiated cellular functions and neuronal activities, while negatively correlated biological processes focused on mitosis and cell cycle phase. Clinical investigation showed that higher FGFR3 expression predicted improved survival for glioma patients, especially in Proneural subtype. Moreover, FGFR3 showed very limited relevance with other receptor tyrosine kinases in glioma at transcriptome level.

**Materials and Methods:**

FGFR3 expression data of glioma was obtained from Chinese Glioma Genome Atlas (CGGA) and TCGA (The Cancer Genome Atlas). In total, RNA sequencing data of 325 glioma samples and mRNA microarray data of 301 samples from CGGA dataset were enrolled into this study. To consolidate the findings that we have revealed in CGGA dataset, RNA-seq data of 672 glioma samples from TCGA dataset were used as a validation cohort. R language was used as the main tool to perform statistical analysis and graphical work.

**Conclusions:**

FGFR3 expression increased in classical and neural subtypes and was associated with differentiated cellular functions. FGFR3 showed very limited correlation with other common receptor tyrosine kinases, and predicted improved survival for glioma patients.

## INTRODUCTION

Glioma, especially glioblastoma, is one cancer type wherein aggressive treatment strategies including surgery, radiotherapy, and chemotherapy provide only palliation [1]. Activation of receptor tyrosine kinases is common in Malignancies [2–7]. Increasing fusions events between different RTKs genes have been revealed as key abnormalities in a range of cancers [8]. Recent researches on glioma have reported that fusion genes, which involve in receptor tyrosine kinases, exert oncogenic or transforming effect [9–11] on glioma. FGFR3-TACC3 fusion gene is one of the most famous fusion events that has been reported and well-studied in glioma. As part of FGFR3-TACC3 fusion gene, TACC3 has been proved to promote tumorigenesis. Singh D. et al. [10] focused on the gained function of fused TACC and found that the fusion events resulted in dislocation of TACC which was followed by abnormal mitosis of tumor cells and aneuploidy. Parker B. C. et al. [11] focused on the dysregulation of expression of the fusion gene through escaping degradation by microRNA-99a.

Active FGF/FGFR signaling axis is indispensable in vascular and skeletal development. An increasing body of work has shown that inhibition of FGFR signaling pathway can lead to anti-proliferative and/or pro-apoptotic effects, warranting further investigation on FGF/FGFR axis as a potential therapeutic target [12]. As a receptor tyrosine kinase, FGFR3 has been reported to have oncogenic effects in mice with mutation of PTEN or K-Ras [13, 14].

However, we also noted some studies which claimed that FGFR3 demonstrated tumor suppressor properties in pancreatic cells with epithelial phenotype [15] and predicted favorable prognosis in bladder cancer when overexpressed [16]. FGFR3 is an essential partner of the transforming FGFR3-TACC3 fusion, but findings of potential tumor suppressive properties in pancreatic cells and bladder cancers suggested that FGFR3 might exhibit different effects in terms of different tissues. Furthermore, the role of FGFR3 in glioma remains undetermined. Taking advantage of CGGA and TCGA projects, we gathered expression data of nearly 1000 glioma samples to take an integrative investigation of FGFR3.

## RESULTS

### FGFR3 expression is consistently up regulated in classical and neural subtypes

**Figure 1:**
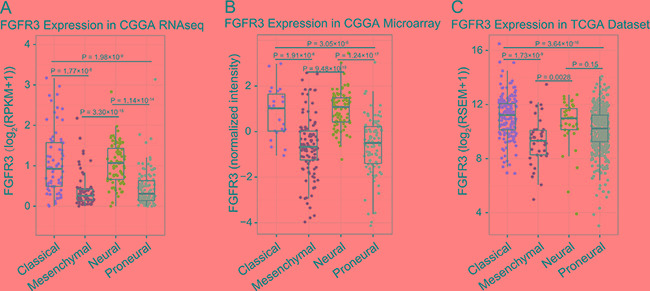
FGFR3 expression in RNA-seq dataset and microarray dataset of CGGA, and RNA-seq dataset in TCGA

**Figure 2:**
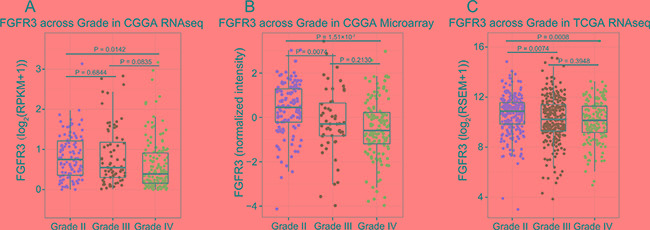
FGFR3 expression pattern across different WHO grades

To get an overview of FGFR3 status, we examined the expression pattern of FGFR3 across four subtypes established by TCGA networks. It turned out that FGFR3 was significantly upregulated in classical and neural subtypes (Figure 1, Student's *t-test*). Additionally, WHO grade was chosen as a second grouping factor. Intriguingly, we revealed that FGFR3 showed very consistent distribution pattern even in different grades across different subtypes (Figure S1, Student's *t-test*). Moreover, FGFR3 tended to express more in lower grade gliomas (Figure 2, Student's *t-test*). To our knowledge, when taking all grades of glioma into account, patients whose tumors belong to neural subtype would get much longer overall survival than patients whose tumors show classical pattern (Figure S2). This result further confounded us about the role of FGFR3 in glioma, reminding us of previous controversial findings in various tumors about FGFR3 [15].

### FGFR3 expression is positively correlated with relatively differentiated cellular function instead of malignant process

**Figure 3:**
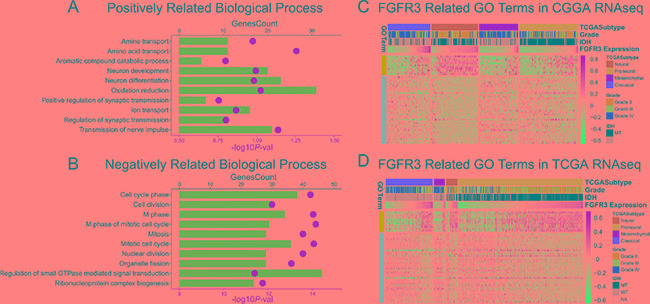
Gene ontology analysis of FGFR3 in RNA-seq dataset of CGGA and TCGA (**A** and **B)** associated GO terms of FGFR3 in CGGA and TCGA dataset, respectively. (**C** and **D)** RPKM and RSEM were log transformed and then mean-centered and normalized before applied to *pheatmap.*

To find out what underlies this expression pattern, we chose classical and neural glioma samples in which FGFR3 expression was significantly upregulated to perform Spearman correlation tests. An *r* value more than 0.3 or less than −0.3 was regarded as significant correlation. CGGA RNA-seq data was used as a training cohort. In classical gliomas 1401 genes were identified while in neural subtype 2085 genes were identified, among which 754 genes were shared by both two subtypes. Gene ontology analysis was performed using DAVID annotation tools (https://david.ncifcrf.gov/). FGFR3 positively correlated genes were most involved in relatively differentiated cellular function, suggesting a neutral or protective role of FGFR3 (Figure 3A). Moreover, FGFR3 was significantly negatively correlated with cell cycle (Figure 3B), which indicated active mitosis and proliferation of tumor cells. The biological processes that FGFR3 involved in reflected its protective property. To get a clear view of the related GO terms of FGFR3, heat map was plotted, together with Mesenchymal and Proneural subtypes which showed relatively lower FGFR3 expression (Figure 3C). Amino acid transport and cell cycle phase were chosen as typical terms for positively and negatively related biological processes, respectively. To our surprise, in both mesenchymal and Proneural subtypes, we observed positive relevance between amino acid transport process and FGFR3 expression, as well as negative relevance between cell cycle phase and FGFR3 expression. This result suggested that FGFR3 was a gene which robustly correlated with relatively differentiated cellular biological process. To validate what we found in CGGA RNA-seq dataset, we performed similar analysis in TCGA RNA-seq dataset, which demonstrated high resemblance to the pattern of CGGA (Figure 3D). To further validate what we found, we sequenced 51 more glioma samples for mRNA expression data. As indicated by the aforementioned results, we applied all the samples, ranging from grade II to grade IV, into gene ontology analysis. In line with the results of CGGA and TCGA RNA-seq data, FGFR3 negatively related genes significantly focused on cell cycle phase and again, mitotic cell cycle ranked first in various biological process terms (Table 1).

**Table 1:** Gene ontology analysis results of an independent cohort of 51 glioma samples

GO Term: biological process	Genes Count	*P*-Value	Benjamini
mitotic cell cycle	92	7.80E-33	1.80E-29
transcription, DNA-templated	208	1.60E-31	1.80E-28
regulation of transcription, DNA-templated	152	1.20E-23	9.40E-21
cell division	66	3.80E-22	2.20E-19
DNA repair	66	1.90E-18	9.00E-16
mitotic nuclear division	45	9.50E-14	3.70E-11
mitotic sister chromatid segregation	14	4.10E-12	1.40E-09
gene expression	99	1.20E-11	3.50E-09

### FGFR3 has very limited association with other RTKs

**Figure 4:**
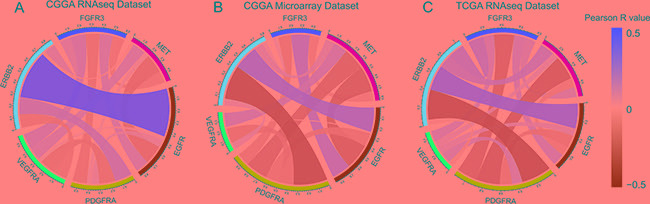
Association between FGFR3 and other RTKs in in RNA-seq dataset and microarray dataset of CGGA, and RNA-seq dataset in TCGA Red ribbons indicate positive correlation of two terms while green ribbons indicate negative correlation. Width of ribbon and scale of colors indicate Correlation coefficient.

FGFR3 originally functions as an RTK and mediates activation of downstream pathways. In addition, it has been revealed to be associated with carcinogenesis. In glioma, alterations in RTKs are essential players in tumorigenesis and progression. Thus, the relationship between common RKTs that were frequently dysregulated in glioma was investigated. Taking all subtypes of glioma as a whole, we observed that FGFR3 expression demonstrated very limited association with other RTKs in all three datasets (Figure 4). Since subtypes might be a confounding factor in analyzing relationship between RKTs, we additionally performed the correlation analysis in isolated subtypes in CGGA (Figure S3A–S3D) and TCGA (Figure S3E–S3H) RNA-seq datasets, which also showed very limited correlation between FGFR3 and other RTKs.

### FGFR3 expression predicts favorable survival for glioma patients

**Figure 5:**
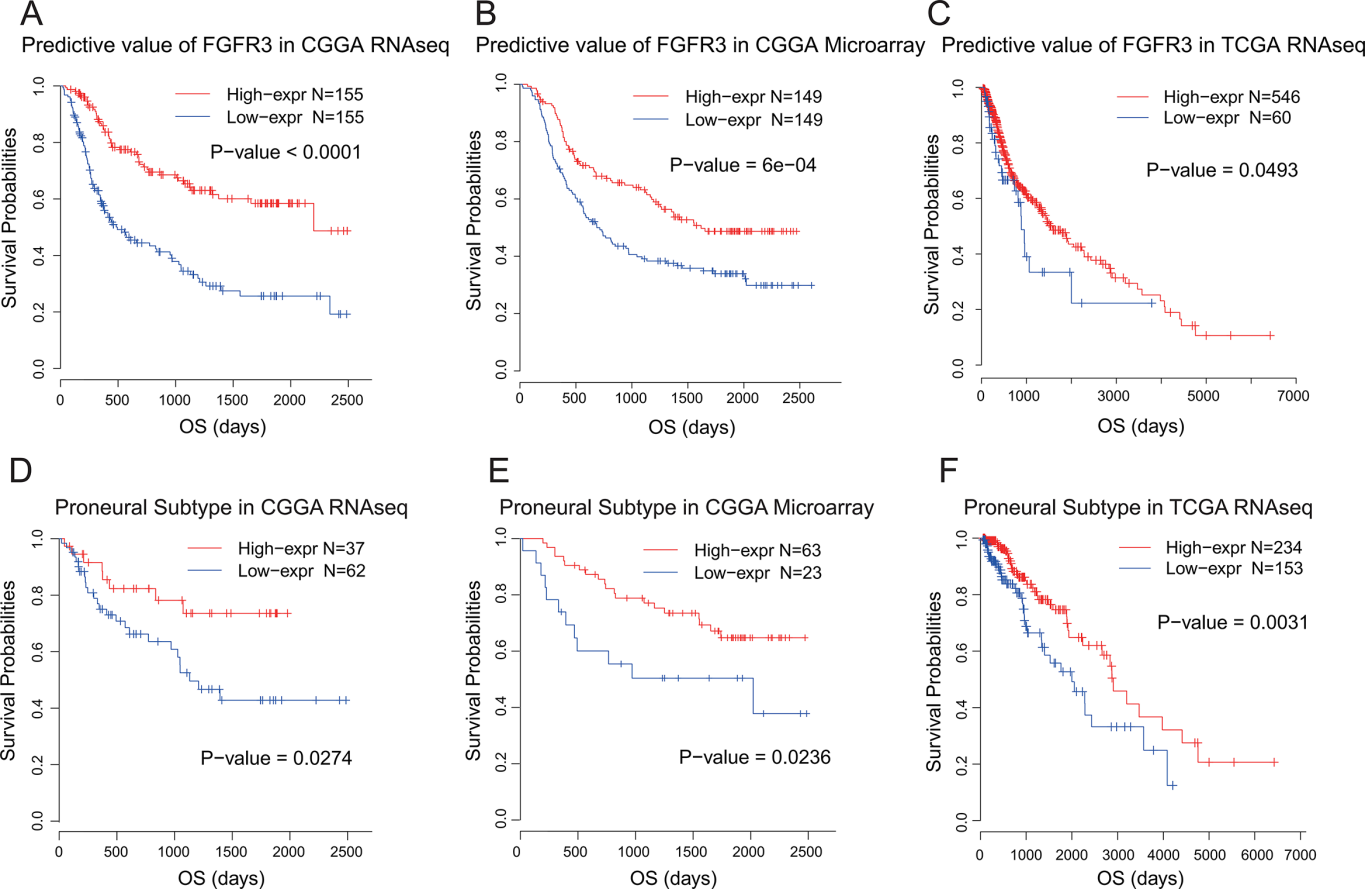
Survival analysis of FGFR3 in glioma and proneural subtype

As FGFR3 showed robust negative relationship with malignant biological process, we additionally interrogated the prognostic value of FGFR3 expression. Kaplan-Meier curves were performed in all three datasets. When taking all gliomas into account, patients who had higher FGFR3 expression in their tumors showed much improved survival than those had lower FGFR3 expression, both in CGGA and TCGA dataset (Log-rank test, Figure 5A–5C). In microarray data and RNA-seq data of CGGA datasets, FGFR3 showed the best predictive value. Multiple Cox proportional hazards analysis was performed, taking multiple clinical and molecular factors into account. As shown in Table 2, FGFR3 was an independent prognostic factor, which further strengthen the protective role of FGFR3 in glioma.

**Table 2:** Multiple variate cox proportional hazards analysis

Terms	Coef	Exp (coef)	Se (coef)	z	*P* value
Age	−0.00854	0.9915	0.00945	−0.9	0.3665
Grade	1.09641	2.99341	0.15522	7.06	1.60 × 10^−12^
Radio	−0.79324	0.45238	0.19804	−4.01	6.20 × 10^−5^
Chemo	−0.47418	0.62239	0.20577	−2.3	0.0212
IDH	−0.90277	0.40544	0.24315	−3.71	0.0002
FGFR3	−0.21726	0.80472	0.10731	−2.02	0.0429

Moreover, we generated Kaplan-Meier curves of FGFR3 in separated subtypes. It turned out that Proneural type was the only subtype in which FGFR3 exhibited consistent prognostic value throughout all three datasets (Log-rank test, Figure 5D–5F). In other subtypes, we failed to identify such consistency throughout three datasets (data not shown). Though FGFR3 showed relatively lower expression pattern in Proneural subtype, it demonstrated the most significant prognostic value.

## DISCUSSION

Genes encoding RTKs are prevalently affected in glioma [17, 18], especially in classical and mesenchymal subtypes. Fusion events of remote chromosome fragments usually occur in malignant tumors where poor stability of chromatin was observed. FGFR3 is a key fusion partner in glioma. We aimed to assess whether FGFR3 could function as an oncogene and promote tumor malignancy. To find out the role of FGFR3 in glioma, we obtained nearly 1000 glioma samples from CGGA and TCGA network.

To our surprise, FGFR3 showed relatively higher expression in classical subtype and neural subtype. To our knowledge, classical subtype and neural subtype are totally distinct subtypes of glioma, which demonstrate a world of difference in biological processes. Consistency of FGFR3 expression in these two subtypes blurred the role of FGFR3. At first, we presumed that FGFR3 may exert different functions in two subtype of tumors. Therefore, we screened for FGFR3 related genes in both Classical and Neural subtypes in CGGA RNA-seq dataset. Spearman correlation was performed. When 0.3 was set as a cutoff for absolute *r* value, 1401 genes were identified as significantly correlated with FGFR3 expression in classical subtype while in neural subtype the number of genes was 2085. However, an overlap of 273 genes was extracted between two sets of correlated genes, which suggested that FGFR3 exert relatively the same function in both two subtypes. These genes were subsequently regarded as FGFR3 robustly related genes. Gene ontology analysis showed these genes are positively correlated with relatively normal cellular functions in central nervous system, such as amino acid transport, neuron development, neuron differentiation and regulation of synaptic transmission. On the other hand, FGFR3 negatively related genes demonstrated tumor related functions, such as mitosis and cell cycle, which indicated frequent cell proliferation. To get further understanding of FGFR3 related function, two typical terms, amino acid transport and cell cycle phase, were chosen and tested in mesenchymal and Proneural subtypes. It turned out that FGFR3 exhibited satisfying consistency in these two subtypes (Figure 3C–3D). These results suggested that, FGFR3 might exert similar function across subtypes on glioma. Additional independent cohort also consolidated the result. Therefore, in line with previous studies, FGFR3 is associated with less malignancy [19, 20].

Moreover, these findings here are in agreement with the role of FGFR3 in gliomas, particularly in the consequences of FRFR3-TACC3 fusion events, where the fusion genes have cancer promoting roles and could be inhibited to gain clinical improvement when treated with JNJ-42756493 [21]. These results further suggested protective role of wild-type FGFR3.

Unlike other RTKs, FGFR3 seemed to function normally even protectively instead of as an oncogene. Pearson analysis showed very limited relationship between FGFR3 expression and other five RTKs. This consolidated the presumption FGFR3 correlated with relatively differentiated cellular function and less malignancy in glioma. Survival analysis provided further evidence to this presumption.

## MATERIALS AND METHODS

FGFR3 expression data of glioma was obtained from CGGA (http://www.cgga.org.cn/) and TCGA (http://cancergenome.nih.gov/). In total, 325 samples of RNA sequencing data and 301 samples of mRNA microarray data from CGGA dataset were enrolled into this study. To consolidate the findings that we have revealed in CGGA dataset, 672 glioma samples of all grade from TCGA dataset were used as a validation cohort. RNA-seq data of CGGA (RPKM value) and TCGA (RSEM value) were log transformed before analysis. R language was used to perform statistical analysis and graphical work. A *p value* less than 0.05 was considered to be statistical significant. This study was approved by the Ethics Committee of Capital Medical University, Beijing, China.

Heatmap was plotted by *pheatmap* package of R developed by Raivo Kolde. Before applied to *pheatmap*, log transformed data of CGGA and TCGA were further centered by mean value of gene (centered by genes) and normalized (Multiply all values in each row of data by a scale factor S so that the sum of the squares of the values in each row is 1.0). Circus plot was achieved with *circlize* package of R developed by Zuguang Gu [22]. Multiple Cox proportional hazards analysis was performed with *coxph* function of *survival* package [23] in R. Student's *t-test* was performed to compare means of two groups of continuous variables.

Survival data of CGGA were collected through follow-up of patients who underwent craniotomy in multi-centers in Beijing as described in our previous study [24]. Survival data of TCGA dataset was obtained from the TCGA website (http://cancergenome.nih.gov/). Written informed consents were obtained from the patients (or their families). Kaplan-Meier curve was generated with *survival* package of R and log-rank test was performed to examine statistical difference of survival between two groups.

## CONCLUSIONS

FGFR3 expression was upregulated in classical and neural subtypes and was associated with relatively differentiated cellular functions. As a receptor tyrosine kinase, FGFR3 showed very limited correlation with other common receptor tyrosine kinases, and predicted improved survival for glioma patients, especially for Proneural subtypes.

## SUPPLEMENTARY MATERIALS FIGURES AND TABLES


