# Supplementary material for: FGFR3, as a receptor tyrosine kinase, is associated with differentiated biological functions and improved survival of glioma patients
Source: Oncotarget. 2016 Nov 5;7(51):84587–93. doi: 10.18632/oncotarget.13139 (PMC5356683; doi:10.18632/oncotarget.13139)
Supplement: Supplementary file 1 [file oncotarget-07-84587-s001.pdf]

FGFR3, as a receptor tyrosine kinase, is associated with differentiated biological functions and improved s-urvival of glioma patients

Supplementary Materials

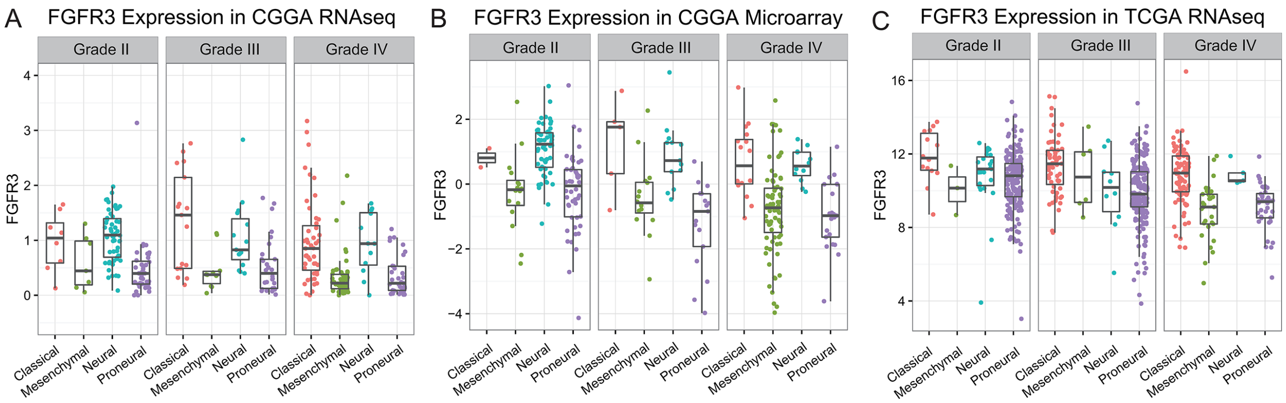

Supplementary Figure S1: FGFR3 expression across subtypes in different WHO grades.

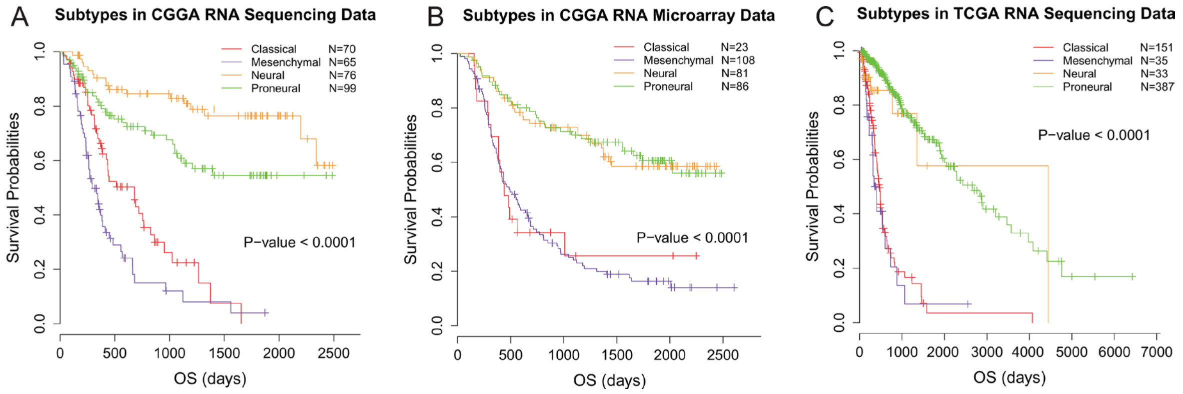

Supplementary Figure S2: Survival analysis of FGFR3 expression between molecular subtypes.

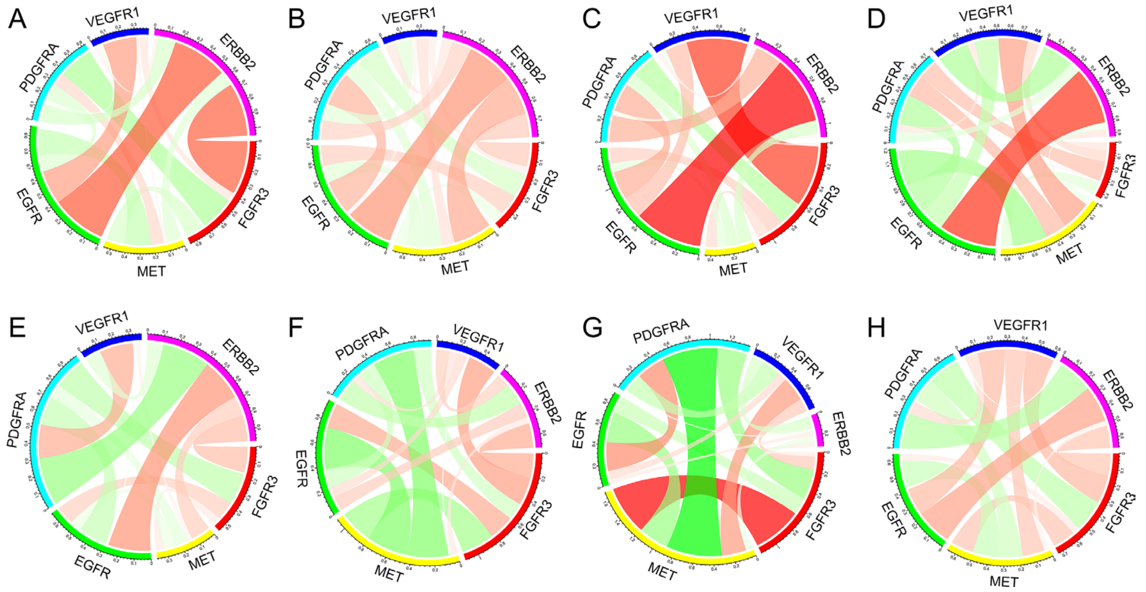

Supplementary Figure S3: Association between FGFR3 and other RTKs in different molecular subtypes. Red ribbons indicate positive correlation of two terms while green ribbons indicate negative correlation. Width of ribbon and scale of colors indicate Correlation coefficient.
